# Influence of Continuous Rotation and Optimal Torque Reverse Kinematics on the Cyclic Fatigue Strength of Endodontic NiTi Clockwise Cutting Rotary Instruments

**DOI:** 10.3390/dj12100317

**Published:** 2024-09-30

**Authors:** Jorge N. R. Martins, Emmanuel J. N. L. Silva, Duarte Marques, Marco A. Versiani

**Affiliations:** 1 Faculdade de Medicina Dentária, Universidade de Lisboa, 1600-277 Lisboa, Portugal; 2 LIBPhys-FCT UID/FIS/04559/2013, 1600-277 Lisboa, Portugal; 3 Grupo de Investigação em Bioquimica e Biologia Oral (GIBBO), Unidade de Investigação em Ciências Orais e Biomédicas (UICOB), 1600-277 Lisboa, Portugal; 4 Centro de Estudos de Medicina Dentária Baseada na Evidência (CEMDBE), 1600-277 Lisboa, Portugal; 5 Department of Endodontics, School of Dentistry, Grande Rio University (UNIGRANRIO), Rio de Janeiro 21210-623, Brazil; 6 Department of Endodontics, Fluminense Federal University, Rio de Janeiro 24220-900, Brazil; 7 Department of Endodontics, Rio de Janeiro University (UERJ), Rio de Janeiro 20550-013, Brazil; 8 Dental Specialty Center, Brazilian Military Police, Belo Horizonte 30350-190, Brazil

**Keywords:** cyclic fatigue, endodontics, kinematics, reciprocation, root canal therapy, scanning electron microscope

## Abstract

**Objectives:** The objective of the present study was to evaluate the cyclic fatigue strength of clockwise cutting rotary endodontic instruments when subjected to two different kinematics: continuous clockwise rotation and clockwise reciprocation movement under optimum torque reverse (OTR) motion. **Methods:** New ProTaper Next X1 (n = 20) and X2 (n = 20) instruments were randomly divided into two subgroups (n = 10) based on kinematics (continuous rotation or OTR). The specimens were tested using a custom-made device with a non-tapered stainless-steel artificial canal measuring 19 mm in length, featuring a 6 mm radius and an 86-degree curvature. All instruments were tested with a lubricant at room temperature until a fracture occurred. The time to fracture and the length of the separated fragment were recorded. Subsequently, the fractured instruments were inspected under a scanning electron microscope for signs of cyclic fatigue failure, plastic deformation, and/or crack propagation. The subgroup comparisons for time to fracture and instrument length were performed using the independent samples *t*-test, with the level of statistical significance set at 0.05. **Results:** When using OTR movement, the ProTaper Next X1 increased the time to fracture from 52.9 to 125.8 s (*p* < 0.001), while the ProTaper Next X2 increased from 45.4 to 66.0 s (*p* < 0.001). No subgroup exhibited plastic deformations, but both showed dimpling marks indicative of cyclic fatigue as the primary mode of failure. Additionally, OTR movement resulted in more metal alloy microcracks. **Conclusions:** The use of OTR motion extended the lifespan of the tested instruments and resulted in a higher number of metal microcracks. This suggests that OTR motion helped to distribute the mechanical stress more evenly across the instrument, thereby relieving localized tension. As a result, it delayed the formation of a single catastrophic crack, enhancing the overall performance of the instruments during the experimental procedures.

## 1. Introduction

The development of nickel–titanium (NiTi) instruments has profoundly transformed root canal treatment, significantly enhancing both its efficiency and effectiveness. Traditional continuous clockwise rotary motion has long been the standard for the mechanical instrumentation of root canals, offering predictable shaping of the canal space. However, using NiTi instruments in a continuous motion also has its drawbacks, such as an increased risk of instrument fracture, canal transportation, and ledge formation, which may affect the overall success of the treatment [1]. In recent years, reciprocation kinematics have emerged as an alternative to continuous rotary motion [2,3]. Reciprocation involves back-and-forth movement with varying angles of rotation (asymmetric oscillatory motion) of NiTi instruments, aimed at enhancing the safety and efficacy of canal preparation [3]. This alternating motion reduces continuous rotational stress, distributing the mechanical load more evenly across the instrument [3]. As a result, reciprocation not only prolongs the instrument’s lifespan but also reduces the likelihood of sudden breakage during use [4,5,6]. Reciprocating motion has also been shown to reduce procedural errors such as ledging and perforation [7]. Although concerns persist regarding its debris removal ability [8,9] and the maintenance of the original canal curvature, reciprocation has demonstrated greater effectiveness compared to continuous rotary motion [9].

Traditionally, reciprocating kinematics involve a counterclockwise cutting direction, requiring specific adjustments in instrument design. However, with adjusted rotation angles, it can result in a clockwise cutting direction, exemplified by the optimum torque reverse (OTR) mechanism. In this mode, the instrument automatically reverses its counterclockwise rotation by 90° upon reaching a predetermined torque threshold. It then alternates with a clockwise cutting motion over 180° until the torque decreases below the set threshold [10], enabling the use of clockwise cutting rotary instruments in a reciprocating motion.

One of the critical factors influencing the selection of NiTi instruments for root canal preparation is their cyclic fatigue strength. Cyclic fatigue is a frequent cause of instrument failure, especially in curved root canals [11,12]. Instruments used in continuous rotary motion are susceptible to cyclic fatigue because of the constant rotational forces applied during canal shaping. This repetitive stress can induce micro-cracks in the metal alloy and eventually lead to instrument fracture, compromising the treatment and elevating the risk of procedural complications [12,13]. In contrast, reciprocation kinematics have been demonstrated to markedly improve the cyclic fatigue strength of endodontic instruments [4]. However, there is limited evidence regarding the viability of using clockwise cutting rotary instruments in a reciprocating clockwise cutting direction. The available evidence [10,14] indicates that rotary instruments could potentially benefit from this motion in terms of cyclic fatigue strength. While not endorsed by manufacturers, adopting this motion, if deemed safe, could enhance clinical practice by extending the lifespan of these instruments and potentially lowering their fracture risk. By comprehensively examining this aspect, it is possible to determine whether clockwise reciprocation can offer a superior alternative to conventional rotary motion in root canal shaping.

Given the gap in the existing literature, this study aimed to assess the cyclic fatigue strength of clockwise cutting rotary instruments under two different kinematic conditions: continuous clockwise rotation and clockwise reciprocation motion (OTR). The null hypothesis to be tested was that there would be no significant differences between the fatigue strengths observed under these two kinematic modes.

## 2. Materials and Methods

Forty new ProTaper Next X1 (PTN X1; n = 20) and X2 (PTN X2; n = 20) clockwise cutting rotary instruments (Dentsply Maillefer, Ballaigues, Switzerland) were randomly selected, inspected for fracture characteristics, and checked macroscopically for major defects, with none discarded.

### 2.1. Cyclic Fatigue Test

To calculate the most adequate sample size (https://sample-size.net/ accessed on 10 January 2024), instruments showing the greatest difference in the initial five tests were considered. With an alpha-level error of 0.05, a statistical power of 80%, and an estimated effect size and standard deviation of 76.6 ± 43.0 (PTN X1) and 20.4 ± 13.6 (PTN X2), the required sample sizes were calculated to be 7 and 9, respectively, to achieve statistical significance. To ensure sufficient statistical power, a final sample size of 10 files was chosen for each group.

The cyclic fatigue strength test followed a previous study setting [14]. It was performed using a custom-built apparatus designed to consistently test an endodontic file rotating freely within a curved canal until separation. The artificial canal was made from a non-tapered stainless-steel tube, measuring 19 mm in length and divided into three sections. The coronal section was a 7 mm straight segment, followed by a 9 mm curved segment with a 6 mm radius and an 86-degree curvature, with the point of maximum stress located in the middle of the curve. The apical section was a 3 mm straight segment. The steel walls were 1.3 mm thick and had an inner diameter of 1.4 mm. The block supporting the artificial canal was attached to a main frame, which also supported a movable holder for the handpiece, ensuring precise and consistent positioning of the files to the same depth within the artificial canal.

The selected files in each group were randomly divided into two subgroups (n = 10) based on the kinematics: continuous rotation or reciprocating OTR motion. In the rotation group, the instruments were operated using an endodontic motor (TriAuto ZX2 motor, J. Morita, Kyoto, Japan) with continuous clockwise rotation at 300 rpm and a torque of 1.5 N·cm, with the auto-stop and auto-reverse functions disabled. In the OTR group, the instruments were used in a reciprocating motion by setting the OTR function to 300 rpm and adjusting the torque limit to the minimum level to produce reciprocating movement without any phase of continuous rotation [10]. All endodontic files were tested with a lubricant at room temperature until they fractured. The time to separation was recorded in seconds using a digital stopwatch, and the recording stopped when breakage was audibly or visually detected. The lengths of the fractured segments were recorded for experimental control purposes.

### 2.2. Scanning Electron Microscopic Analysis

Using a conventional scanning electron microscope (SEM) (S-2400, Hitachi, Tokyo, Japan), the fractured tips and surfaces of the instruments were carefully inspected at magnifications of ×100 and ×150. The SEM was operated with an acceleration voltage of 20 kV, a filament current of 3.1 A, and a working distance of 20 mm. The surfaces were examined for characteristics indicative of cyclic fatigue failure, including plastic deformation (unwinding or twisting) and crack propagation (chevron pattern) as previously described [13,15].

### 2.3. Statistical Analysis

The time to fracture was expressed as mean values with standard deviations. To ensure the validity of the statistical analysis, the distribution of the results was assessed and confirmed to follow a Gaussian (normal) distribution using the Shapiro–Wilk test. Given this normal distribution, comparisons between the sub-groups were made using the independent samples *t*-test, allowing for an accurate assessment of differences between groups. The threshold for statistical significance was set at 0.05 (SPSS v.22 for Windows; IBM SPSS Statistics, Chicago, IL, USA).

## 3. Results

**Figure 1:**
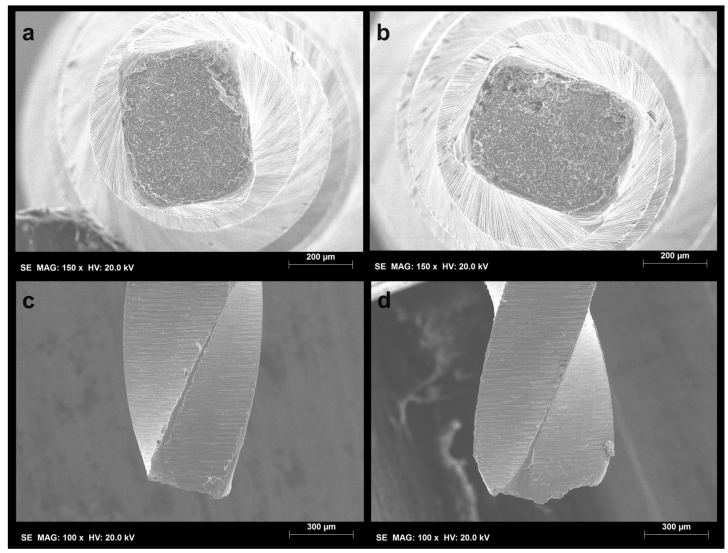
Representative photos of the separated surfaces of ProTaper Next X1 files: (**a**) axial view of the separated surface from the rotation group; (**b**) axial view of the separated surface from the OTR group; (**c**) lateral view of the separated area from the rotation group; (**d**) lateral view of the separated area from the OTR group. The dimpling areas visible on the whole instrument’s axial view surfaces and the absence of plastic deformations on the tips indicate features consistent with cyclic fatigue failure.

**Figure 2:**
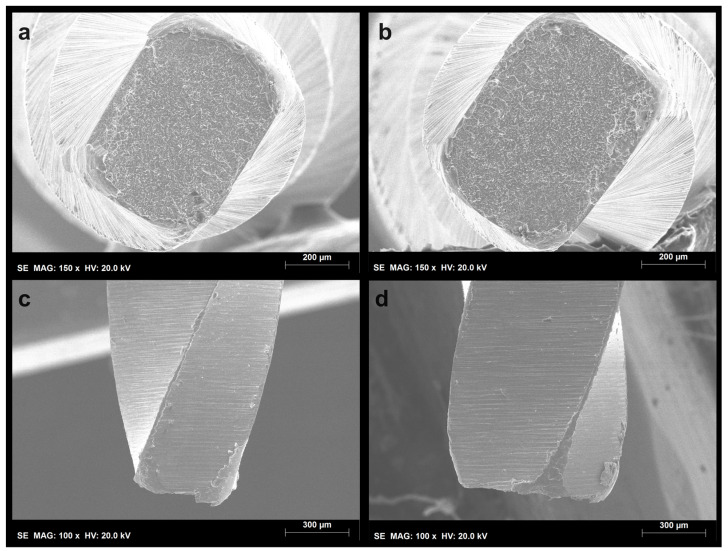
Representative photos of the fractured surfaces of the ProTaper Next X2 instruments: (**a**) axial view of the fractured surface from the rotation group; (**b**) axial view of the fractured surface from the OTR group; (**c**) lateral view of the separated area from the rotation group; (**d**) lateral view of the separated area from the OTR group. The dimpling areas visible on the whole instrument’s axial view surfaces and the absence of plastic deformations on the tips indicate features consistent with cyclic fatigue failure.

**Figure 3:**
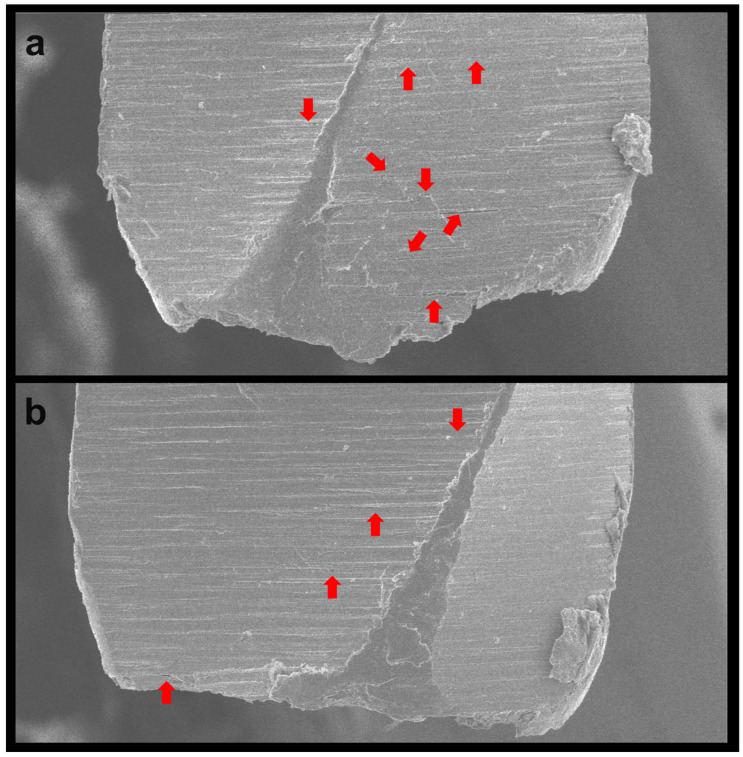
Representative images of the OTR groups showing metal alloy microcracks (red arrows) in both the ProTaper Next X1 (**a**) and ProTaper Next X2 (**b**) instruments.

Both the PTN X1 and X2 instruments demonstrated a significant increase in their time to fracture when activated in the OTR motion. For the PTN X1 instruments, the time to fracture increased from 52.9 s to 125.8 s (*p* < 0.001), while the PTN X2 instruments showed an increase from 45.4 s to 66.0 s (*p* < 0.001) (Table 1). No plastic deformation of the tips was observed in any of the subgroups. However, all groups, regardless of the type of motion (rotation or OTR), exhibited extensive dimpling areas characteristic of cyclic fatigue failure (Figure 1 and Figure 2). Additionally, the fractured surfaces of the instruments subjected to OTR motion revealed a higher number of metal microcracks (Figure 3).

## 4. Discussion

A previous editorial [16] questioned the clinical relevance of in vitro cyclic fatigue tests of rotary and reciprocating endodontic instruments using predefined artificial canals. While it is true that this test by itself provides little meaning regarding a particular instrument’s strength, and this is why the multimethod approach has been advocated to overcome this methodological limitation [17], it is also true that cyclic fatigue tests using predefined artificial canals have historically provided significant understanding on how to improve the instruments’ strength under specific testing conditions that may indeed be relevant to clinics. Previous works assessing endodontic files for cyclic fatigue have shown that material composition, heat treatments, file design, and motion type significantly impact their durability and clinical performance [1,2,3,4,11]. NiTi files exhibit superior cyclic fatigue resistance compared to stainless-steel files, particularly when enhanced through heat treatment. The design of the files, including their taper, cross-sectional shape, and tip configuration, also plays a critical role, with variable taper and triangular cross-section designs showing enhanced resistance [1,2,3,4,11]. Additionally, reciprocation motion, which involves alternating movements, has been found to reduce the incidence of cyclic fatigue failure compared to continuous rotary motion, due to decreased stress concentration on the files [2,4]. The curvature and radius of root canals further influence cyclic fatigue life, with more pronounced curvatures increasing the risk of failure [11]. These findings have direct clinical implications, guiding the selection of instruments for specific endodontic procedures to minimize the risk of file separation and improve treatment outcomes [5].

The present study used the ProTaper Next X1 and X2 instruments due to their wide acceptance by clinicians, and the artificial canal had a 6 mm radius and an 86-degree curvature [14] in order to take the instruments to an extreme curvature. Additionally, the auto-stop and auto-reverse functions were disabled for the OTR motion so that its reciprocating kinematics (the variable of interest) would not be influenced by any predetermined torque threshold. This was performed to ensure similar conditions for comparison with continuous rotation, which was also not torque-dependent. The results reveal a significant improvement in the cyclic fatigue strength of PTN X1 and X2 instruments under clockwise reciprocation motion (OTR) compared to continuous clockwise rotary motion. These findings support the hypothesis that using clockwise reciprocating kinematics effectively extends the lifespan of endodontic instruments by reducing cyclic fatigue, thereby lowering the risk of instrument fracture during clinical use. As a result, the null hypothesis was rejected.

The significant extension in time to fracture observed in both the PTN X1 and X2 instruments under OTR motion is consistent with findings from previous studies [10,14], underscoring the advantages of reciprocating motion in increasing cyclic fatigue resistance. Specifically, PTN X1 instruments demonstrated an increase from 52.9 s to 125.8 s, while PTN X2 instruments increased from 45.4 s to 66.0 s (Table 1). A previous study by Pedullà et al. [10] tested five different clockwise cutting endodontic instruments, also comparing conventional clockwise movement and OTR motion (a clockwise reciprocation kinematic), and reported a time to fracture increase between 35.3% and 131.4%. Another posterior study [14] also compared five distinct files under the two described motions and described a mean increase ranging from 52.1% to 156.7%. These results suggest that OTR reciprocating motion effectively distributes mechanical stresses more uniformly across the instrument, thereby mitigating the formation of microcracks that typically precede instrument failure [13].

Scanning electron microscope analysis revealed extensive dimpling areas on the axial fractured surfaces in both motion types, characteristic of cyclic fatigue as the primary mode of failure [18,19]. Moreover, no plastic deformation was observed, confirming that the fracture did not result from torsional failure. However, instruments subjected to OTR motion exhibited a higher incidence of metal microcracks. While initially counterintuitive, this finding suggests that while OTR motion extends the instrument’s overall lifespan by reducing continuous stress, it also leads to the formation of numerous smaller cracks rather than a single catastrophic failure point. This distribution of micro-damage could indicate a more controlled and predictable wear process, potentially facilitating better clinical monitoring and timely replacement of instruments before catastrophic failure occurs.

The OTR motion starts with continuous clockwise rotation until reaching a specific predefined trigger torque, at which point asymmetric oscillatory motion begins. In this study, the trigger torque was set at its minimum value (0.2 N), following protocols from prior research [10,14], to eliminate the initial continuous rotation phase of OTR motion and standardize settings across both experimental groups. This approach isolated reciprocating movement as the primary variable of interest. The test temperature was maintained at room temperature (20 °C), although some studies argue that using body temperature (typically 36 °C) better simulates clinical conditions [20,21]. However, several authors have demonstrated that neither intra-canal temperature [22,23] nor NiTi instruments exiting the root canal space [20] reach body temperature. This discrepancy has led previous studies to question the appropriateness of using body temperature as a standard for testing NiTi instruments [22]. In response, the present study adhered to international guidelines from the American Society for Testing and Materials [24] for testing superelastic NiTi materials and considered the proposed revisions to ISO 3630-1 [25], opting to conduct tests at room temperature. The observed longer time to fracture of the smaller-sized instruments (PTN X1) and the consistent fracture patterns of the separated instruments are consistent with findings from previous research [26,27], thereby supporting the methodological rigor of the current study protocol.

The enhanced cyclic fatigue strength observed with OTR motion has significant clinical implications. In root canal treatment, navigating curved root canals always carries the risk of instrument fracture. Implementing OTR reciprocating motion could amplify the benefits of reciprocating kinematics in clockwise cutting instruments, potentially reducing procedural complications such as canal transportation, ledging, and perforation, while simultaneously prolonging the lifespan of instruments [9]. Additionally, the ability to employ existing clockwise cutting rotary files in OTR reciprocating motion without requiring specialized counterclockwise cutting instruments or dedicated endodontic motors could streamline clinical workflows and lower costs [14]. This adaptability could make OTR motion more practical in everyday dental practice, providing a safer and more efficient alternative to traditional continuous clockwise cutting rotary motion. However, there are also drawbacks, such as the potential for the apical extrusion of intracanal material, which may influence periapical inflammation and post-operative pain [28].

While this study supports the advantages of OTR motion, it also has limitations. The experimental setup using a custom-made device and artificial canals may not fully replicate natural root canal complexities. Another issue that may raise methodological doubts, considering the variability of previous studies, is the use of different lubricants or different testing temperatures. Regarding the latter, it has been widely debated, with a recent publication showing that NiTi instruments are sensitive to temperature changes caused by irrigation solutions and intraoral temperatures, making it difficult to define an ideal temperature for testing [22]. It would be expected that an increase in the testing temperature would decrease the time to fracture for both kinematics, since the Protaper Next has an austenitic start temperature around −5 °C and an austenitic finish near 48 °C, which means the acquisition of austenitic characteristics happens exactly in the service range temperature between 20 °C and 36 °C [29]. Consequently, validating these findings in clinical settings is not feasible, and therefore, conducting multimethod research [17] to evaluate the impact of clockwise reciprocation motion on other stresses like torsion, as well as on outcomes such as cutting ability and debris extrusion, would be valuable. Multimethod research has been noted for its ability to provide a more comprehensive understanding by using qualitative and quantitative methodologies that complement each other and enhance the ability to address research questions, contributing to the acquisition of valuable insights for clinical practice [17,30]. Testing with other torque settings, and using a wider range of endodontic instruments, with different designs and heat treatments, could further validate these findings. Shaping ability testing using natural teeth and micro-Ct technology would also provide more insight into the comparison of both kinematics. A comprehensive assessment addressing these aspects would offer a more complete understanding of the clinical effectiveness and safety of reciprocating clockwise cutting kinematics in clockwise cutting instruments.

## 5. Conclusions

The present study underscores that reciprocating clockwise cutting motion (OTR) improved the cyclic fatigue strength of PTN X1 and X2 instruments compared to continuous clockwise cutting rotary motion. The reciprocating motion led to a greater occurrence of metal alloy microcracks, suggesting reduced mechanical stress and a delay in reaching a single catastrophic failure point. Reciprocating clockwise cutting motion used on clockwise rotary instruments may enhance their clinical strength.

## Figures and Tables

**Table 1:** Mechanical performance reporting time to fracture and fragment length (mean ± standard deviation) and its percentage mean increase when alternating from continuous rotation to OTR kinematics.

Instrument	Rotation Group (n = 10)	OTR Group (n = 10)	% of Increase	*p*-Value
Time to Fracture (Seconds) *				
ProTaper Next X1	52.9 ± 5.0	125.8 ± 16.9	137.8%	<0.001
ProTaper Next X2	45.4 ± 7.2	66.0 ± 9.1	45.4%	<0.001
**Fragment Length (Millimetres)**				
ProTaper Next X1	7.4 ± 0.3	7.6 ± 0.4	2.7%	0.421
ProTaper Next X2	7.5 ± 0.3	7.4 ± 0.5	−1.3%	0.761

* The number of cycles is not presented since the rpm for both kinematics is equally 300 rpm.

## Data Availability

Data are contained within the article.
